# Research on the Relationship of the Organization Capacity and Job Satisfaction in Nursing Homes

**DOI:** 10.1093/geroni/igaa057.595

**Published:** 2020-12-16

**Authors:** Lulu Liao, Huijing Chen, Yinan Zhao, Hongting Ning, Hui Feng

**Affiliations:** 1 central south university, Changsha, China; 2 Central South University, Changsha, China

## Abstract

Objective: To explore the occurrence of organization capacity and to examine the relationships among the organization capacity and job satisfaction in nursing homes in China. Methods: A total of 577 participants in sixteen nursing homes in Hunan province of China were enrolled using a cluster randomized controlled trial. Staff job satisfaction was measured by Minnesota Satisfaction Questionnaire(MSQ) in short form, nursing homes’ organization capacity was assessed by Shortell’s Organization and Management Survey. One-way analysis of variance examined differences between variables. The relationship and correlation of organization capability, MSQ total score and scores in each dimension was analyzed. Results: Overall score of nursing homes’ organization capacity was 99.5±8.6(level ranking, 21–105), and MSQ score was 78.2±8.2 (level ranking, 20–100). Staff job satisfaction levels were significantly different among nursing staffs with different levels of organization capability (F=9.40, P＜0.001). Correlation analysis showed that organization capability level was positively correlated with staff job satisfaction (r=0.52, P< 0.001). And Correlation analysis showed that Working conditions (r = 0.46, P < 0.001), Leaders (r = 0.46, P < 0.001), Responsibility (r = 0.51, P < 0.001) and External reward (r= 0.42, P < 0.001) were positively associated with organization capability. Conclusion: The organization capability can be improved by active staff job satisfaction. But the job satisfaction of nursing staffs was at a moderate level. Thus effective measures such as creating a supportive work conditions, appointing suitable leaders, enhancing responsibilities and providing external reward should be considered by nursing managers to promote organization capability in nursing homes.

